# School health policies and their implementation during the COVID-19 pandemic in the Philippines

**DOI:** 10.1186/s41182-024-00659-4

**Published:** 2024-12-23

**Authors:** Mikaela B. Salanguit, Marian Danille C. Santillan, Ernesto R. Gregorio, Crystal Amiel M. Estrada, Fumiko Shibuya, Akihiro Nishio, Jun Kobayashi

**Affiliations:** 1 Department of Health Promotion and Education, College of Public Health, University of the Philippines Manila, 625, Pedro Gil Street, Ermita, Manila Philippines; 2 Department of Environmental and Occupational Health, College of Public Health, University of the Philippines Manila, 625 Pedro Gil St., Manila, Philippines; 3 Department of Global Health, Graduate School of Health Sciences, University of the Ryukyus, Nishihara, Japan; 4 Japanese Consortium for Global School Health Research, Nishihara, Japan

**Keywords:** School health, COVID-19, Pandemic, Policy, Policy implementation

## Abstract

**Introduction:**

The COVID-19 pandemic has severely impacted the health and education of learners globally. However, there is a lack of information on enablers and barriers to the implementation of comprehensive school health policies during the pandemic.

**Methods:**

This study utilized a case study design, and was conducted in the Division of City Schools of Navotas. A desk review of relevant school health policies released at the national level by the Department of Education and Department of Health during the pandemic was performed. The collected policies were classified if they were related to the Preparedness/Prevention Phase, Early Phase Response, and Chronic Phase Response. Focus group discussions and key a informant interview were conducted to determine the enablers and barriers in implementing school health programs during the pandemic. The identified themes were created deductively by using categories from Whitman’s Wheel of Factors influencing Implementation of Policy and Practice.

**Results:**

Policies under the Preparedness/Prevention Phase focused on providing a comprehensive healthy school environment for learners, including Water, Sanitation, and Hygiene (WASH) in Schools. Early Phase Response policies included school closures and public health guidelines to prevent further spread of disease. The policies under the Chronic Phase/Response notably included guidelines for implementation of vaccination days and reopening of classes. Most of the factors identified were categorized under Whitman’s wheel of factors.

**Conclusions:**

Despite COVID-19 restrictions, health policy implementation continued because of the collaborations, innovations, and leadership of various stakeholders. The vaccination of the public, including the pediatric population was implemented through the collaboration of various agencies. WASH in Schools was evident in policies regarding the guidelines on prevention of the spread of disease. The data provided in this study will serve as a guide to address the barriers and further strengthen the implementation of these policies.

## Introduction

The COVID-19 pandemic is a global public health crisis, causing a distressing socioeconomic impact. As of May 14, 2023, there had been over 766 million confirmed cases of COVID-19 globally, 6 million of which led to death [1]. In the Philippines as of December 10, 2023, the total number of confirmed cases was 4,127,856 with 66,779 deaths [2]. In terms of its mental health effects, studies have shown that the general population had increased levels of distress and symptoms of depression and anxiety during the early stages of the pandemic [3, 4]. Preventive measures implemented to control the virus included social distancing, wearing of face masks, and lockdowns. The situation forced the previous face-to-face activities to transition to working and learning remotely and online.

In particular, the COVID-19 pandemic had severely impacted the health and education of learners globally. In fact, the pandemic is expected to slow down the attainment of the Sustainable Development Goals No. 3 (Good Health and Well-being) and No. 4 (Quality Education). At the peak of the pandemic in 2020, more than 1.6 billion learners from 180 countries were affected by these school closures [5]. This further exacerbated the gap between low- and high-income families, i.e., children from high-income families had the means to sustain an online mode of learning while children from low-income families struggled due to limited access to digital devices, unstable or unavailable internet connection, and a home environment that was not conducive to online learning [6, 7].

The effects of prolonged school closures due to the pandemic were not only observed in terms of poor educational outcomes, but also in children’s poor mental health. Due to school closures, malnourished children from low-income families did not have access to school-based feeding program which contributed to poorer physical and mental health outcomes [6, 7]. Physical distancing and community quarantine measures were implemented to further curb the spread of the virus. However, this also limited the physical activities of children, leading to sedentary lifestyles and potential weight gain. The greater amount of time spent indoors and the shift to online learning during the pandemic also increased the chances of longer screen time for children, a practice which has been linked to childhood obesity [8].

School closures have also significantly affected the well-being and mental health of school children. Anxiety, depression, and psychological distress have been reported [9, 10], and children with attention deficit hyperactivity disorder have been shown to exhibit worsening behavior during the pandemic [11]. In addition, school closures disrupted the important link between child abuse victims and school personnel, leading to decreased reports of child abuse cases [12]. In the Philippines, school closures were implemented at the national level starting in March 2020^.^ and schools were reopened only in August 2022 [13]. This was reportedly one of the longest pandemic-induced school closures in the world, lasting for 2 and a half years. It has been reported that prolonged school closures have led to children becoming more vulnerable to mental health issues and, in some cases, sexual abuse of children at home where the perpetrators are their relatives [13]. However, as the schools reopened in 2022, classroom protocols included measures such as infection control, appropriate ventilation, physical distancing, handwashing facilities, dispersed school attendance through varying schedules for each grade level, and hygiene education for the learners [14–17].

The Global School Health Initiative proposed by the WHO in 1995 emphasized the importance of schools as places that can function as facilitators of health-related activities in the community [18]. In 2000, FRESH: Focusing Resources on Effective School Health was proposed as a comprehensive school health framework that emphasizes not only health services and health education, but also environmental development and policy formulation [19]. In the Philippines, a comprehensive approach was introduced in 1987 with the development of a manual integrating various school health activities [20]. The “Nutrition Programs (SHNP) for the Achievement of the Education for All (EFA) and the Millennium Development Goals (MDGs)” was published in 2011, clearly stating comprehensive school health as a national program [21]. In the context of the COVID-19 pandemic, the comprehensive school health approach would include: (1) health services, such as testing and treatment of COVID-19; (2) health education for infection control; (3) a supportive environment for setting up hand-washing facilities; (4) avoiding discrimination and prejudice toward infected children; and (5) a management system to implement these measures. Therefore, implementing a comprehensive school health approach is key to continuing face-to-face implementation of classes while implementing infection control and addressing its psychological impact on learners.

Tomokawa and colleagues [22] identified factors related to the implementation of comprehensive school health policies in studies in Thailand and Laos. These findings which were identified before the COVID-19 pandemic have been presented during the WHO Technical Meeting on School Health in 2015. A study in Nepal [23] revealed that a lack of coordination between stakeholders, limited training opportunities, lack of resources, and uncertainty about the sustainability of the program were barriers to school health and nutrition program implementation. In the Philippines, a study found that hygiene promotion activities and hygiene management topics are discussed as needed and integrated in some subjects as personalized practical lessons. A lack of running water and soap in hand washing areas and a lack of a harmonized lesson plan for health promotion were identified as barriers [24].

Despite the widely reported negative effects of the COVID-19 pandemic on children’s health, there is a dearth of information on enablers of and barriers to the implementation of comprehensive school health policies during the pandemic. While a study was conducted in Zambales, Philippines, on the education response to the COVID-19 pandemic which included implementation challenges, this study focused more on the implementation of policies and guidelines related to the learner’s educational outcomes rather than health [25]. Studies on the Philippine government’s policy response to COVID-19 only included public health safety measures, and were not specific to school health [26–29]. Thus, this study aimed to describe the school health-related policies that were implemented, their enablers and the implementation barriers during the COVID-19 pandemic. Describing the various school health-related policies from this review will provide a comprehensive reference for an evidence-based response to a future pandemic especially the enablers and barriers to policy implementation.

## Methods

### Policy reviews

A literature review of relevant school health-related policies by relevant government agencies before and during the pandemic, especially those issued by the Department of Education (DepEd) and the Department of Health (DOH) was conducted. Documents were selected based on the US CDC’s definition of a policy [30]. The list of policies available in the DepEd [31] and DOH [32] online databases were used as the references for which policies were reviewed. School health policies issued from the year 2000 until May 5, 2023, when the WHO declared an end to COVID-19 as a public health emergency, were included in the review [33]. When certain policies were unavailable from these online database, other online sources were used, such as The Official Gazette, the official journal or repository of public policies of the Republic of the Philippines [34]. The keywords used include health, programs, hygiene, COVID-19, Mental Health, Food, Nutrition, Comprehensive, Inclusive, Sustainable development. All publicly available policies and selected policies related to school health, including Republic Acts (RA), Implementing Rules and Regulations (IRR), Administrative Orders (AO), Proclamations, Memorandums, and Department Circulars (DC) were checked and reviewed. The salient features of the policies such as their title, date of issuance, organization responsible, and main content, were summarized in a matrix.

### Study settings and participants

This study utilized a case study design [35] and was conducted in Navotas City, a city in the National Capital Region. This city was purposively selected based on its urban location as well as being part of the National Capital Region which is one of the top regions in terms of active COVID-19 cases as of January 2022 [36]. The Schools Division Office in Navotas City was selected since this is the local office of the Department of Education in charge of implementing and coordinating policies for public schools in Navotas City [37]. The largest school in terms of student population in the district was likewise purposively selected.

### Interviews

Two focus group discussions (FGDs) and one key informant interview (KII) were conducted. The participants in both FGDs were based on who were available at the time of the visit. In the first FGD, the participants were seven (7) School Division Office (SDO) representatives. These representatives included all the school health personnel (a dentist, and nurses) who are providing health services to the learners and teachers. No physician participated in the FGD since there was no item or position for such in the SDO at the time of data collection. The second FGD included four (4) teachers who taught Reading, Math and Science. Teachers also held other administrative positions such as the coordinator for the Alternative Learning System and Program Coordinator for Nestle (Physical Fitness). The KII was done with the school principal. A topic guide was developed for the interviews that focused on how the policies were implemented during the COVID-19 pandemic. In particular, the interviews focused on identifying the enablers of and barriers to school health policy implementation Table 1.

**Table 1:** Background of participants of the focus group discussions and key informant interview

	Participant	Description
Focus group discussion 1 (SDO)	Participant 1	Chief of school governance & operations division
	Participant 2	Nurse II
	Participant 3	Nurse II
	Participant 4	Dentist II
	Participant 5	Dentist II
	Participant 6	Nurse II
	Participant 7	Nurse II
Focus Group Discussion 2 (School)	Participant 1	Alternative learning system coordinator
	Participant 2	School teacher for reading
	Participant 3	School teacher for math and science
	Participant 4	Program Coordinator for Nestle (physical fitness programs)
Key Informant Interview	Participant	School principal

### Data analysis

#### Policy review

The policies that are directly or indirectly related to school health were collected and classified according to whether they were related to the preparedness/prevention phase, early phase response, or chronic phase response. Policies issued before the pandemic were considered part of the preparedness/prevention phase. Policies categorized under the early phase response are from the first detected case of COVID-19 in the Philippines (January 30, 2020) [38] to the first detected omicron variant in the Philippines (December 14, 2021) [39]. Policies are then considered to be under the chronic phase response if they were issued from December 15, 2021 onward.

#### Qualitative data

The audio recordings of the key informant interview and focus group discussions were transcribed into Word documents. NVivo version 12 was used to generate codes and themes from the data. The themes found were created deductively by using categories from Whitman’s Wheel of Factors influencing implementation of policy and practice [40].

According to Whitman, there are 12 factors influencing the implementation of policy and practice. These factors are namely: (1) vision and concept/ international and national guidelines; (2) dedicated time and resources; (3) stakeholder ownership and participation; (4) team training and ongoing coaching/learning community; (5) cross-sector collaboration; (6) champions and leaders at all levels; (7) data-driven planning and decision-making; (8) administrative and management support; (9) adapting to local concerns; (10) attention to external forces; (11) critical mass and supportive norms; and (12) stage of readiness.

Permission to conduct the study was granted by the SDO of Navotas and the school principal. Ethical review and exemption were granted by the University of the Philippines Manila Ethics Review Board (UPM-REB).

## Results

### Review of major school health-related policies issued by government agencies before and during the pandemic

#### Preparedness/prevention phase (before January 30, 2020)

Annex 1 shows the relevant school health policies released at the national level by the Department of Education and the Department of Health during the pandemic. Several major school health-related policies were issued by the national government before the pandemic. One is a Department Order from the Department of Education while two are Republic Act or Laws. These are the Department Order No. 028 (*OK sa DepEd)* [41], Republic Act 11036 (*The Mental Health Act*) [42], and the Republic Act 11223 (*Universal Health Care Act*) [43]. Notably, several Department Memoranda provide guidelines on the implementation of the School-based Immunization Program such as the DepEd Memorandum No. 82, s. 2015 [44], DepEd Memorandum No. 128, s. 2016 [45], and DOH’s Department Memorandum No. 2015–0238 [46]. All of these policies contained provisions related to the implementation of school health programs.

In 2018, the DepEd issued *OK sa DepEd*, which aims to provide all learners and DepEd personnel with a comprehensive school health and nutrition program and services for healthier behaviors that will contribute to better learning outcomes. *Ok sa DepEd* is a convergence of the department’s six flagship programs, namely, School-Based Feeding Program (SBFP) complemented by other nutrition support programs; Medical, Dental, and Nursing Services, including the School Dental Health Care Program (SDHCP); Water, Sanitation, and Hygiene (WASH) in Schools (WinS) Program; Adolescent Reproductive Health; National Drug Education Program supported by comprehensive tobacco control; and the School Mental Health Program [41]. A policy and guidelines were developed for the implementation of the WinS program in 2016 [47]. This document contains basic requirements and standards on water, sanitation, hygiene, health education, and deworming, as well as the roles and responsibilities of the different offices of DepEd, the schools, and partner organizations. Monitoring and evaluation of the program implementation is also clearly stated in the guidelines.

The Mental Health Act, or the Republic Act 11036 was approved in 2018. One of the objectives of this Act is to incorporate strategies that promote mental health in educational institutions, the workplace, and communities. In line with this, Section 24 of the Act requires institutions to develop policies and interventions for learners, educators, and others to increase awareness on mental health, provide support and services for those at risk, and facilitate access, including the referral of individuals with mental health conditions to treatment and psychosocial support [42]. To further support the Republic Act, the implementing rules and regulations of Republic Act 11036 also direct educational institutions to ensure the systematic procedure for counseling and referral of learners in public schools and guide all public schools and governance levels in the implementation of counseling and referral [48].

The Republic Act 11223, also known as the Universal Health Care (UHC) Act, was approved in February 2019. Section 30 of the UHC Act mandates that all schools under DepEd be designated as healthy settings based on standards set by the DOH and DepEd. Furthermore, DepEd is mandated to formulate programs and modules on health literacy and rights that will be integrated into existing school curricula. The objective is to intensify the fight against both communicable and noncommunicable diseases through a healthy lifestyle and behavioral risk factors approach. Furthermore, Section 30.6 of the UHC Implementing Rules and Regulations specifies that schools designated as healthy settings will be based on the following components, which are essentially adopted from the WHO Health Promoting School Framework: health school policies, school environment (physical and social), health skills and education, links with parents and community, and access to health services [43].

There are also policies in place for the implementation of school-based immunization. Although vaccination is not considered as a requirement, learners enrolled in Grade 1 and Grade 7 would have their vaccination records screened. Learners with zero or incomplete doses of recommended immunization would be provided with a free vaccination service administered by the health center and schools, provided that the parent or guardian consented to the student being vaccinated. If the student has missed the scheduled routine immunization by the schools, they will be accompanied by the school nurse and referred to the nearest Rural Health Unit. The learners will not be suspended or reprimanded if they have zero or 1 dose of vaccination, or if their guardian has refused their vaccination. Information and education campaign (IEC) materials, and advocacy and communication plans should also be developed [44–46].

#### Early phase response—January 30, 2020 to December 14, 2021

A total of 40 policies were included and analyzed as part of the early phase response. Policies with multiple amendments, such as the Omnibus Guidelines on the Implementation of Community Quarantine in the Philippines, were counted as a single policy.

During the pandemic, DepEd and DOH issued a number of Department Memoranda, Circulars and Administrative Orders directly related to the implementation of hand hygiene, mental and psychosocial health, oral health, minimum public health standards, vaccination, school-based feeding, and basic education continuity plans related to COVID-19. Additionally, several policies have been issued regarding COVID-19 vaccination. This includes policies such as *Bayanihan, Bakunahan* National COVID-19 Vaccination Days, which included guidelines on making vaccination more accessible to the general public [49], as well as guidelines on the vaccination of the pediatric population, specifically those that are 12–17 years old [50, 51].

#### Chronic phase response (on or after December 15, 2021)

Policies during the chronic phase of the COVID-19 pandemic focused on the vaccination of the general population and the provision of guidelines in public areas and schools in preparation for the return of face-to-face classes. Several policies were issued regarding the repeated implementation of the *Bayanihan, Bakunahan* National COVID-19 vaccination days on different dates [52, 53]. During the reopening of face-to-face classes, policies that were issued to maintain public health standards included the establishment of WASH facilities and supplies in strategic locations and the provision of IEC materials on hygiene practices and respiratory etiquette [54–56]. As with the Guidelines on the Implementation of School-Based Immunization, advocacy campaigns were to be conducted to encourage teachers, school personnel, and learners to be vaccinated against COVID-19.

Pursuant to the UHC Law, the DOH, DepEd, Department of Social Welfare and Development (DSWD), Commission on Higher Education (CHED), Legal Education Board (LEB), Technical Education and Skills Development Authority (TESDA), and the Department of the Interior and Local Government (DILG) issued Joint AO No. 2022, 0001 dated March 14, 2022, entitled Guidelines on Health Settings Framework in Learning Institutions. The order aims to provide a framework, through the coordination of the aforementioned government agencies, for the establishment and/or strengthening of healthy learning institutions across life stages in the Philippines [57].

### Enablers and barriers of the implementation of school health policies

A total of 89 codes, 6 categories, and 14 sub-categories were generated from the FGD and KII. These codes were then recategorized into themes according to Whitman’s wheel of factors influencing the implementation of policy and practice.

#### Vision and concept

At the beginning of the pandemic, the previous face-to-face implementation of classes was no longer allowed to prevent the transmission of COVID-19. Classes were then conducted online, with the supplemental physical aid of modules distributed to the learners through the parents’ school visits. As schools abruptly shifted to online classes, school health leaders needed to focus on their clear vision of school health. This was translated through the collaboration of external partners such as parents, local government units, and sponsors.

Excellent leadership was needed and commended by the personnel and teachers. They were encouraged to persevere in innovating strategies to ensure that quality education was provided to all learners amidst the pandemic. In Whitman’s wheel of factors influencing the implementation of policy and practice [40], a strong concept to motivate people into action is key to changing practice. This was also evident in the FGD with the Schools Division Office (SDO) staff and the interview with the school principal where it was observed that participants were knowledgeable about the school health policies being implemented as well as its purpose. A common statement from the participants was that they were striving to implement these policies since they had a clear vision of school health.

*The folic acid is for females. DepEd Order 2017, part of the Adolescent Reproductive Health program, menstruating females. This is still for the students, in preparation for them since they’re developing. They’re prone to iron deficiency because of their menstruation. Besides this, there is also nutritional imbalance in our adolescents because as they grow, they become weak because of the food they eat. They are prone to IDA, so we supplement them with folic acid. This also affects their outputs in their studies, they might become lethargic.(Participant 2, FGD SDO)*

On the other hand, misconceptions from parents were identified as a barrier to the implementation of school health programs. Since program implementation was adjusted in line with physical distancing guidelines, teachers relied on the cooperation of parents in the delivery of services and programs. For example, teachers relied on parents receiving and administering deworming tablets to their children. However, not all parents accepted the deworming tablets nor did they allow their children to take them. The parents’ lack of confidence in these services was attributed to the controversial implementation of the (Dengvaxia) dengue vaccination drive among learners, which was discontinued due to safety concerns a few years back. In some instances, parents were too preoccupied with their jobs, to avail of services for their children.

*Because before, when we distributed deworming tablets, they didn't accept it, the parents rejected it. One of the reasons is the dengvaxia, (Participant 1, FGD SDO)*

#### Dedicated time and resources

Although the teachers received only a few reports of student mental health problems, they still raised concerns over the inadequate number of mental health professionals to whom they can refer their learners.

*But for mental health, that’s one of the challenges for us since we don’t have a psychiatrist, that’s who we’re looking for. (Participant 1, FGD SDO)*

The respondents also identified a lack of manpower as a barrier to school health program implementation. At the time of the interviews, there were only six (6) members of the school health team who catered to the needs of the 50,000 learners in the city. This problem was exacerbated during the pandemic when the health personnel of the local government unit were not able to provide assistance to the school health team because of their COVID-19-related tasks at the city. This lack of manpower severely limited the ability of the school health team to provide medical services to the entire student population of the city.

*They [LGU] sent us a letter asking if we could distribute because they can’t do it themselves because they’re assigned to the vaccination site and isolation center. So there's a lack of manpower. (Participant 1, FGD SDO)*

*because we only have limited manpower. So as much as we want to cover it all, we really cannot, and with 50,000 learners and we only have 6 para-medical and dental personnel that’s why… (Participant 1, FGD SDO)*

The division could not address the problem related to lack of medical doctor at the SDO, since local guidelines do not indicate the allocation of government-hired medical doctors for medium-sized divisions such as Navotas City.

*There is no item for a doctor at a medium-sized division; we’re only medium. (Participant 1, FGD SDO)*

In addition to the lack of medical personnel, there is a lack of guidance counselors assigned to schools. There was only one guidance counselor in the entire school division, who was assigned to one school. The remaining schools in the division only have guidance teachers or guidance coordinators.

*There is only one guidance counselor here in SDO that is registered in a High School. (Participant 2, FGD SDO)*

In handling programs at the regional, city, and school levels, there is a designated focal person for each health program. Additionally, in each school, a coordinator for each grade level is assigned to focus on each class at their respective grade level. During the interviews, some participants shared that there are difficulties in continuing the implementation of health programs when focal persons leave and need to be replaced. Focal persons assigned to a specific program are already familiar with its implementation, but the challenge arises when they are reassigned and the newly appointed focal person of the programs would need more time to learn.

*One of the barriers would be whenever they would change the focal person..whenever they would change the designated focal person. (Participant 3, FGD SDO).*

Further compounding the challenges in program implementation is the lack of equipment and facilities, which existed prior to the pandemic. One of the most cited examples during the focus group discussion was the lack of dental facilities. Since there was no dental facility available, they were not able to perform dental procedures and had to refer the learners to another facility.

*Just like what we mentioned earlier, we lack medical and dental facilities. That's why, even if they wanted to perform procedures that they need to perform, oral treatment, they can’t do so because we don’t have the facilities. (Participant 1, FGD SDO)*

In addition to medical and dental facilities, the need for more handwashing facilities to support group handwashing activities was identified.

*We need more handwashing for one is to one population, because in WINS, that’s our weakest area, because we’re three stars already, but we’re lacking in group handwashing. (Participant, KII Principal)*

On the other hand, as classes had to transition to an online setup, informants mentioned that learners experienced issues such as having unstable internet connection and a lack of gadgets to continue their studies. In terms of their ability to use technology, teachers informally classified their learners as either of the following in terms of the use of technology: “high tech”, “low tech”, or “no tech”. Those categorized as “no tech” were provided with printed modules that the teachers would retrieved after the learners had accomplished them.

Aside from unstable or limited internet connectivity issues, teachers also expressed concerns for learners who were categorized as low tech, or those who could not go online. In one of the focus group discussions, participants wanted to determine the underlying cause for the learners’ inability to join the online classes. Teachers expressed concern about possible cases of abuse at home as the real cause of learners’ inability to join online classes.

*And the identification of the teachers with students who are suffering from anxiety or are victims of abuse, it’s difficult, it’s really difficult. It’s a very big challenge to identify, and if there are children suffering from child abuse, we wouldn’t know, the school can’t identify them, the teacher can’t identify them. Since a lot of those that experienced this are the ones that can’t go online. (Participant 1, FGD SDO)*

Teachers also experienced technological challenges due to a lack of devices available to support their online teaching activities. They experienced difficulties using laptop computers issued by the Department of Education since the computers lagged when sharing material online and were slow to boot and operate.

*We experienced screen sharing and the slides did not go to the next slide because it is slow. (Participant 2, FGD Teachers)*

Teachers also expressed their concerns about their workload when the pandemic began. It was more challenging for the teachers to practice work–life balance when the remote learning setup was established.

*There really is a lot more during the pandemic, Some may think (otherwise)… but there really is more work. (*Participant 4, FGD SDO)

Aside from their regular teaching load, teachers are burdened with the need to organize more events and attend more meetings and simultaneous events. The move to a virtual learning and working space also meant that teachers had to follow multiple channels of communication, such as different online chats or message groups for specific programs or activities.

*...since sometimes, the activities happen simultaneously, there are overlaps in schedule. Sometimes, teachers would attend three [meetings] at a time. (Participant 1, FGD SDO)*

#### Stakeholder ownership and participation

A recurring phrase behind innovation for the continued implementation of school health programs was the need to continue providing these services for the learners, and a reflection to stay relevant and still accomplish something during the pandemic when all face-to-face activities were not allowed.

*We can’t leave them and not care, right? (Participant 2, FGD Teachers)*

*Actually, we thought about how we could be relevant, since we have a saying: strive for relevance…We are active regarding mental health, because we’re pressured. Since we need to accomplish something. (Participant 4, FGD SDO)*

During health program implementation, parents are usually the touchpoint for reaching the learners, especially during the pandemic when classes are usually held online. Thus, it was mentioned that a challenge experienced during program implementation was the inability of parents to visit the school due to work.

*The only thing we noted was the support and participation of parents, since usually they’re at work. So sometimes, they are unable to pick up the food for feeding, that’s where parenting comes in. (Participant, KII Principal)*

#### Team training and ongoing coaching

For mental health, a program called *Helping Fellows*, was implemented. Through this program, select teachers, especially those who served as guidance teachers, were trained in providing support for the learners’ mental health services.

*So we identified helping fellows from the schools, so they will provide what they learn during the training focusing on the mental health of the learners. These helping fellows, guidance counselors, others were health coordinators, it depends on the principal who they want to send or designate as participants. Some are DRRM coordinators, Supreme Student Government Advisers, so the 3 that undergo the training with them and some of our SGOD personnel undergo training. That’s seven sessions. (Participant 1, FGD SDO)*

#### Cross-sector collaboration

As schools had to transition to an online mode of learning, it is evident that this new setup has challenged the implementation of health programs for reaching learners. Collaborations among the local government unit (LGU), the SDO, non-governmental organizations (NGOs), health centers, schools, and the private sector, as well as support from the DOH, were commonly mentioned as part of the implementation of these programs.

*For us, we rarely encounter problems regarding that because it’s easy to collaborate with us. (Participant 1, FGD SDO)*

Additionally, through the School-based Feeding Program (SBFP), bread and milk which were procured by the SDO through a bidding process were distributed to houses with the help of the LGU and parent volunteers.

*For each distribution, the medical team gets split up [is divided and assigned], they visit all the schools during the distribution to the parents during the pandemic. (Participant, KII Principal)*

As limited face-to-face classes slowly began, partnerships were formed for the supply of hygiene kits on each classroom table for the learners. Compounding the lack of mental health professionals is the lack of a referral system and clear guidelines for referral. To address this challenge, informal referral systems in the form of partnerships with NGOs were established. An example of this would be the Helping Fellows program, which was implemented through a partnership with a mental health institution.

Although the guidance counselor or guidance teacher may be equipped with knowledge in psychological first aid, the learner, teacher, or non-teaching staff will be referred if ever they need professional help.

*Clinic teachers attend seminars regarding PFA, referral systems, we really prepare them so they’re ready. (Participant 1, FGD SDO)*

At all levels, each stakeholder exemplified their commitment to continuing education and the implementation of health programs at home. Starting from DepEd and their respective LGUs to each school principal and health program coordinator, parents were also highly involved in ensuring that their children were still learning from their classes at home. Each level requires a committed champion in successful school health implementation, especially during the pandemic.

Despite this, it is evident that teachers were then forced to learn urgently how to use the new online platforms for classes and activities. Everything that was previously given physically and onsite, was transformed into its online counterpart. There is also an additional barrier to the availability of the internet both at school and at home for both teachers and learners.

*That is where we were pressured, how do you plan to continue what you’ve been doing this pandemic? The pandemic at first made it seem like we won’t have work anymore, but in fact, our workload increased heavily since we needed to adapt. (Participant 4, FGD SDO)*

#### Data-driven planning and decision-making

Through conferences, each school was able to learn from each other, as they all shared their reports on the success of program implementation. For example, one of the best practices shared during the conference was the high distribution rate of modules and continuation of the health programs in school. As there was a sudden shift to online modalities for classes, the school shared how they asked for support from the parents for the distribution of the physical copies of the modules, along with the distribution of deworming tablets and folic acid in school. This was then implemented in other schools as well to ensure the continuity of education and health programs from home.

As mentioned previously, the respondents identified the need for more structured guidance on mental health support for learners. The respondents stressed the importance of and their desire to have a guide or a manual for teachers to follow should their learners need mental health support or treatment; however, their ability to develop the manual is limited.

*…but we don’t know how to develop it [manual]. (Participant 1, FGD SDO)*

Compounding the lack of mental health professionals is the lack of a referral system and clear guidelines for referral. To address this challenge, informal referral systems in the form of partnerships with NGOs were established. The participants expressed their need for more collaborations with mental health institutions for the referral of learners and faculty and a content guide to be incorporated into their classes.

*For the referral system, there’s none (no training for it) yet. (Participant 2, FGD SDO)*

#### Administrative and management support

Another component indicated as central to the concept of Whitman’s wheel of factors influencing the implementation of policy and practice is the participation of stakeholders such as the teachers, community members, parents, and learners [40]. This is observed through the mention of qualities relating to good governance during the interview. These qualities were mentioned as facilitators of health policy implementation in their region.

*Quick approval, quick implementation. (Participant 1, FGD SDO)*

Ownership and participation of the teachers were noticeable through the distribution of tasks and open communication in place in most of the implementation strategies.

*I had a substitute, we alternated because I’m assigned in the morning, and my substitute is for the afternoon. So when I go home, someone will replace me. (Participant 2, FGD SDO)*

*Yes (there is a focal person in each school for each part), one for NDEP, one for feeding, and one for dental, and so on. (Participant 1, FGD SD*O)

As learners are mostly at home, teachers are not able to see and identify learners who may be suffering from anxiety, COVID-19, or abuse. Sometimes when learners are absent from class, it is automatically assumed that they do not have an available device or internet, or that they may be sick from COVID-19. In these instances, there is a need for more guidance as teachers lose their relationships with their learners due to the online modality.

*It’s hard for the teachers to identify their students who suffer from anxiety or if they are victims of abuse. That’s a very big challenge to identify who even if we say that there are kids that suffer from child abuse, we don’t know, the school and the teacher can’t identify. As of the moment, there are so many who have problems that aren’t able to go online. So these students that are not able to go online, the presumption is always they don’t have gadgets. The possible reason why they really are not online is because they do have problems that are not captured. Especially if the perpetrator is within the family. Even if teachers undergo training, but then since you don’t see them face to face unlike before where you can see if a student isolates themselves, you can ask them if they have problems. But now, you can’t, if a student doesn’t go online, a teacher just assumes they have no internet, no funds for internet load, no gadget. It doesn’t reach the aspect of considering if the student has other personal problems or if they have the COVID virus. (Participant 1, FGD SDO)*

### Attention to external forces

During the FGD with school health personnel, participants mentioned that regular evaluation of school health programs through a Program Implementation Review (PIR) is performed quarterly in the division and then at the regional office of DepEd. During the review, a representative is required to present the situation in their school and what they have accomplished. Quantitative measures such as the percentage of learners with normal nutritional status, the number of deworming tablets or food packs that were distributed, and liquidation reports were used to describe the accomplishments.

*Actually there’s also the Program Implementation Review (PIR) that we accomplish. This is done quarterly in the division based on an operational plan…In the region also, we also have PIR with each coordinator. (Participant 1, FGD SDO)*

Assessment tools are cascaded from the DepEd regional office to the division offices to ensure the quality of program implementation. Reports are then accompanied by data acquired from these tools as various means of verification, such as the date received, signature of the recipient, and photo or video proof of the activities undertaken, e.g., the child taking the medicine, including the possible reasons why the products were rejected. These are determined through interviews with parents through an assessment tool provided by the division and regional offices.

*… the parents, they don’t want to accept so those are being monitored. How many accepted and how many rejected, how many were distributed to the parents. This is part of monitoring. (Participant 1, FGD SDO)*

*There’s a tool cascaded from the division, there are also questions for the parents from the region. (Participant, KII Principal)*

The monitoring of school health programs is usually done by the principal of the school, with the help of the master teachers and the grade-level leaders, as documented through an accomplishment report. SDO officials, such as a doctor and nurses also visit the schools to evaluate the program. Partner NGOs have also been mentioned for having their own monitoring for school health program implementation.

*They [principals] are the ones who constantly monitor even before the pandemic. (Participant 1, FGD SDO)*

*Yes, there’s also monitoring by the SDO officials on health. The doctor and nurse visits to evaluate if the program is followed. (Participant 3, FGD Teachers)*

*Our monitoring is separate, since we have school-based monitoring with master teachers as well as GLL as the ones in charge. (Participant, KII Principal)*

The informants also mentioned that the compliance of schools with established quality assurance systems such as ISO certification helped maintain school health project implementation. Strict quality control is also implemented with “inspectorate” teams in charge of checking for molds, expiration dates, and cleanliness of food packs.

*I told them, what will be the basis for your performance? Since we are ISO certified, and they have a process that requires deliverables and as we compare that to their CRF, this is their accomplishments last year…(Participant 1, FGD SDO)*

The LGU also checks the compliance of schools with water quality standards, waste segregation, number of available hand-washing facilities, and quality of comfort rooms. Reward systems such as the “Region for Best Implementer of WASH” and the awarding of the Seal of Effective School Governance, which gave recognition to the efforts of schools on continued implementation, were also mentioned.

*At WinS [Wash in Schools], water testing is included, also segregation (of waste), even the quality of the comfort rooms is tested. (Participant 2, FGD Teachers)*

#### Stage of readiness

To fulfill the health programs, the interviewees mentioned strategies to overcome the challenges posed by the pandemic. A common attitude observed among the responses of the policy implementers was the willingness to adapt to the situation.

*How do we continue our services for our learners, also with the teachers since all of them are at home…So ma’am said we should think of a way to continue the program during the pandemic, we can’t go house to house, think of a way.. We should be flexible.*

*(Participant 1, FGD SDO)*

*We only have a certain budget allocated for this, but it’s not that big that’s why we find ways through barangay assistance. (Participant, KII Principal)*

The interviews revealed that during the pandemic, schools focused on the SBFP, deworming, distribution of folic acid, oral health, and mental health. These programs were cascaded through the SDO Online Aralan, an online platform where schools are informed about the guidelines for continuation of the program through an online setup. To further facilitate information dissemination and communication among the focal persons, group chats were made for each of the programs. *SDO Online Aralan* was a result of the desire of the SDO to continue to be relevant despite the pandemic. *SDO Online Kalusugan* (Health Online) and Online Dental Consultation were eventually added.

*Since all of them are at home, we came up with SDO Online Aralan para sa Kalusugan and Dental Consultation. (Participant 4, FGD SDO)*

A common strategy mentioned was the integration of school health policy with planned school activities, such as the distribution of folic acid, deworming tablets and IEC during the scheduled distribution of school modules. Physical exercise was also integrated as a regular activity before the class starts for the day. School health and nutrition topics are also integrated into school modules when appropriate.

*…every morning before they take their attendance, they do the project feng check in…For us in lesson planning and in teaching, the teachers contextualize everything according to true scenarios. We contextualize it based on the situation in Navotas, what they do in their houses when it comes to COVID. The teachers contextualize, sometimes as early as the review, COVID is introduced. (Participant 2, FGD Teachers)*

Despite the pandemic restrictions, the participants of the study viewed this to have a positive impact on school health program implementation.

*Another good thing that happened during the pandemic/the good effects of the pandemic to us, was when it comes to implementation of health programs, for example, when we conduct health talks, since there are no children in school, we do it on an online platform, actually I also learned about it a little bit. (Participant 4, FGD SDO)*

*It seems the programs even increased during the pandemic. Nothing was stopped. It intensified and was modified due to distance learning. (Participant 3, FGD Teachers)*

## Discussion

This study aimed to describe school health-related policies during the COVID-19 pandemic and the enablers and barriers encountered during the implementation of such policies during the pandemic. The COVID-19 pandemic has drastically changed the way the educational system was designed and implemented. Consequently, health promoting strategies in schools needed to be responsive and required more technical and on-ground support.

Due to the community quarantine implemented in the Philippines, the education sector was forced to shift to alternative learning modalities, ranging from purely online to modular or a combination of both. This also affected the implementation of school health policies. However, school health policies established before the pandemic helped mitigate the effects of the pandemic since the existing policies such as WASH and vaccinations in schools only needed to be recalibrated given the COVID-19 pandemic as a context.

Certain strategies included in the comprehensive WASH in schools program in the Philippines developed in 2016 needed to be modified due to the closure of schools and the transition to online classes. The study participants reported that the importance of handwashing was being integrated into lessons and the group handwashing strategy was also implemented through online classes, where the teacher and learners washed their hands in their own homes, at the same time. As schools gradually returned to face-to-face classes, WASH remained as an important strategy to prevent further spread of COVID-19 [54, 58, 59]. However, the need for more handwashing facilities in schools still remained as a challenge.

Vaccination is also important in ensuring a child’s health and wellbeing [60]. This is especially true during the COVID-19 pandemic, when infection rates in this demographic increased [61]. To increase vaccination rates in school-aged children and adolescents, school-based vaccination was implemented [62–65]. In February 2022, Filipino children aged 5 to 11 years were allowed to receive COVID-19 vaccinations [66]. With this, the DOH coordinated with the DepEd and LGUs to facilitate school-based COVID-19 vaccination in both private and public schools [67]. This strategy was also used in Seattle, Washington, where they were able to vaccinate 50,000 learners in 106 schools through strategic messaging, school-located vaccination clinics, and school-led community engagement [68].

During the conduct of this study in April 2022, it was reported by the World Health Organization that nearly 66% of Filipinos are fully vaccinated [69]. Despite the evidence for the importance of vaccination among children [70], vaccine hesitancy remains a challenge globally [71] and in the Philippines, where vaccine hesitancy has increased due to a dengue vaccine controversy. Additionally, challenges still arise in reaching the most vulnerable population, and local government units were urged to provide vaccination centers closer to their homes.

Vaccination efforts during the COVID-19 pandemic affected the implementation of other school health policies. LGU resources were redirected to the vaccination services and isolation centers, which led to DepEd school health leaders mobilizing and leading the deworming strategies instead.

Adapting to local concerns was identified as an enabler in health program implementation. The integration of health programs in various school activities or routines, the integration of school health topics in education modules, and the distribution of deworming tablets and folic acid along with the distribution of modules, was reported. This strategy was also implemented in other parts of the Philippines [24] as well as internationally [72]. Similar to the reports of the participants in this study, there were no clear guidelines on the number of hours of instruction for hygiene promotion, and the topic was usually included as a practical lesson. A meta-synthesis [73] also revealed that school-based policies were commonly unclear, and stakeholders did not view these policies as compulsory. Therefore, good governance, such as open communication and proper cascading of policies, was reported to assist in the implementation of health policies. Studies have also identified communication, specifically that of properly introducing and informing stakeholders of policy changes and the reasons behind them, as an enabler of policy implementation.

The commendable dedication of the school health personnel was also highlighted during the interviews. The respondents reported concern for the learners’ well-being as a reason for the implementation of school health programs. Previous studies shared similar findings [73, 74], but the results of another study [24], which found that teacher participants complied with STH interventions because of a mandate and not to eliminate a health problem, was contradictory. A factor that could have affected this would be the knowledge of this study’s participants on the policies and the reasons for their implementation.

The KII and FGDs identified the presence of quality assurance systems as one of the reasons for the success of school health programs, which is consistent with that of previous studies highlighting s consistent compliance review and award systems as enablers, and the lack of accountability was seen as a barrier to policy implementation [22, 73, 75]. Training, webinars, and orientations as a strategy for the implementation of school health programs was also identified by the participants in the current study. This finding is supported by other literature indicating the importance of training as an enabler [23, 72] as well as the lack of training as a barrier to program implementation [73].

Consistent with the findings of previous studies done in Lao PDR and Indonesia [22], the study also emphasized the importance of partnerships with LGUs, NGOs, and private companies in the continuous implementation of health programs during the pandemic. [74, 76]. Through these partnerships, health promotion and capacity building may increase. In the Philippines setting, a study on disease control interventions for school-aged children [24] revealed that the call for partnerships with companies that provided in-kind donations for the treatment of STH has successfully reached the schools included in the study population. Another study [73] reported collaboration between different agencies, such as education and health agencies, as an enabler of policy implementation as well as compliance. The same study [73] has found that costs associated with the installation of facilities were reported as a barrier to implementation. The participants of this study, however, were provided with the handwashing facilities through the aforementioned collaborations, specifically those with NGOs. Labana et al. [24] noted that although WASH areas were available in schools, some of these areas did not have available running water and most did not have soap available.

Another factor mentioned in both the FGDs and the KII was the support provided by the parents for implementation. In a study by Ayalew et al. [77], encouragement from family members was significantly associated with annual ivermectin treatment adherence. Grady et al. [72] also reported the influence of child and parent preferences on the implementation of healthy eating policies and physical activity practices. However, there are still cases of resistance from parents due to controversies about the complications caused by Dengvaxia, a dengue vaccine, as was already observed in a 2019 study [24]. The participants of this study addressed this through the provision of IEC materials with deworming tablets, as well as through monitoring compliance with pictures or videos taken by parents.

In a meta-synthesis [73], five studies were able to identify the home environment such as lack of support from parents as a barrier to policy implementation. Although the context for these studies was healthy food and beverage policy implementation, these factors were also identified by the respondents of the FGD and KII as barriers. Unsafe home environments in an online classroom setting were viewed as a barrier to education and a barrier for learners to access health services. The lack of confidence of parents in medicine leading to their lack of support for its administration at home has also been a barrier to health program implementation, specifically deworming.

## Limitations

The findings of the study must be interpreted while taking into account several limitations. The FGD and KII were conducted in one school and one SDO only. These settings were chosen for the study due to their urban location and number of active COVID-19 cases at that time. Additionally, the FGD and KII were conducted during the early phase response to the pandemic and when the school was still implementing an online mode of learning. This probably has an effect on the comprehensiveness of our study as we were not able to get the perceptions of the learners and their parents. In line with this, the focus was given to the policy analysis, with the data from the FGD and KII providing insight into the real-life implementation of these policies.

## Conclusion

By analyzing school health policies before and during the COVID-19 pandemic and conducting interviews with school health leaders, the enablers of and barriers to the implementation of school health-related policies during the pandemic were identified. Most identified factors involved in the implementation of school policies during the COVID-19 pandemic were similar to those in the Whitman framework. Through these identified factors, there has been success in the implementation of a comprehensive school health despite COVID-19 restrictions. Water, sanitation, and hygiene (WASH) and vaccination in schools were evidently shown to be essential components of school health. Successful health policy implementation continued because of the collaborations, innovations, and leadership of the Department of Education, school heads, and faculty. The data provided in this study will serve as a guide to address these barriers and further strengthen the implementation of these policies. We also recommend getting the perceptions and experiences of the learners and their parents relative to school health policy implementation during the pandemic. Through this study, similar institutions and stakeholders may be guided in the implementation of the policies and best practices presented by the respondents of this study.

## Data Availability

The data used in this study are available upon reasonable request.

## Annex

See Table 2.

**Annex 1:** Relevant school health policies released before and during the COVID-19 pandemic

Document	Education/health	Title	Date	Organization responsible
Analysis sheet 1 reviewed policy documents of the timeline of preparedness/prevention (phase #1)				
Republic act	Education			
	Health	Republic Act No. 11036 Mental Health Act (An Act Establishing a National Mental Health Policy for the Purpose of Enhancing the Delivery of Integrated Mental Health Services, Promoting and Protecting the Rights of Persons Utilizing Psychosocial Health Services, Appropriating Funds Therefor and Other Purposes)	2018/6/20	National government (passed by the house of representatives and senate)
		Republic Act No. 11223 entitled “An Act Instituting Universal Health Care for All Filipinos, Prescribing Reforms in the Health Care System, and Appropriating Funds Therefor”	2018/6/23	Department of health
		Republic Act 11148 Kalusugan at Nutrisyon ng Mag-Nanay Act (An Act Scaling Up The National And Local Health And Nutrition Programs Through A Strengthened Integrated Strategy For Maternal, Neonatal, Child Health And Nutrition In The First One Thousand (1000) Days Of Life, Appropriating Funds Therefor And For Other Purposes)	2018/11/29	17th congress
		Republic Act No. 10354, or the Responsible Parenthood and Reproductive Health Act of 2012	2012/7/23	15th congress
		Republic Act No. 9165 “Comprehensive Dangerous Drugs Act Of 2002”	2002/6/7	12th congress
		Republic Act No. 10611 Food Safety Act of 2013	2013/8/23	15th congress
		Republic Act No. 11037 “Masustansyang Pagkain para sa Batang Pilipino Act”, An Act Institutionalizing a National Feeding Program for Undernourished Children in Public Day Care, Kindergarten, and Elementary Schools to Combat Hunger and Undernutrition Among Filipino Children and Appropriating Funds Therefor	2018/7/24	17th congress
		Republic Act 11358 or “National Vision Screening Act.”	2019/7/31	17th congress
		Republic Act No. 11332 An Act Providing Policies and Prescribing Procedures on Surveillance and Response to Notifiable Diseases, Epidemics, and Health Events of Public Health Concern, and Appropriating Funds Therefor, Repealing for the Purpose Act No. 3573, Otherwise Known as the “Law on Reporting of Communicable Diseases”	2018/7/23	17th congress
Implementing rules and regulations	Education	–	–	–
	Health	Implementing Rules and Regulations of Republic Act No. 11036, otherwise known as the Mental Health Act	2019/1/22	Department of health
		Implementing Rules and Regulations of Republic Act No. 11223, otherwise known as the Universal Health Care Act	2019/10/10	Department of health
Administrative order	Education	–	–	–
	Health	Administrative Order No. 2016–0039 Revised Operational Framework for a Comprehensive National Mental Health Program	2016/10/28	Department of health
Department order	Education	DepEd Order No. 13, s. 2017 Policy and Guidelines on Healthy Food and Beverage Choices in Schools and in DepEd Offices	2017/3/14	Department of education
		DepEd Order 10, s. 2016 Policy and Guidelines for the Comprehensive Water, Sanitation, and Hygiene in Schools (Wins) Program	2016/2/19	Department of education
		DepEd Order 40, s. 2012: DepEd Child Protection Policy	2012/5/14	Department of education
		DepEd Order 32, s. 2017: Gender Responsive Basic Education Policy	2017/6/29	Department of education
		DepEd Memorandum No. 083, s. 2019 Oplan Kalusugan sa DepEd One Health Week	2019/7/9	Department of education
		DepEd Order No. 30, S. 2018—Preventive Drug Education Program Policy For Curriculum And Instruction	2018/7/12	Department of education
		DepEd Order 52, s. 2008 Compliance with DepEd Policies on Food Safety in Schools	7/2/2008	Department of education
		DepEd Order 8, s.2007 Revised Implementing Guidelines on the Operation and Management of School Canteens in Public Elementary and Secondary Schools	2/6/2007	Department of education
		DepEd Order 28, s. 2018 Policy and Guidelines on Oplan Kalusugan sa Department of Education	2018/7/6	Department of education
		D.O 31, s. 2018: Policy Guidelines on the Implementation of Comprehensive Sexuality Education	2018/7/13	Department of education
		DepEd Order 65, s. 2009 Implementation of Essential Health Care Program (EHCP) for the School Children	6/22/2009	Department of education
		DepEd Order 87, s. 2012 Guidelines on the Implementation of the HNC-Funded School-Based Feeding Program (SBFP)	12/18/2012	Department of education
		DepEd Order 51, s. 2016 Implementation of the School-Based Feeding Program for School Year 2016–2017	2016/6/29	Department of education
		DepEd Order No. 033, s. 2019 Implementing Guidelines on the Comprehensive Oral Health Program of the Department of Education	2019/12/9	Department of education
		DepEd Order No. 036, s. 2019 Guidelines on the Implementation of School-Based Feeding Program-Milk Feeding Program Component	2019/12/13	Department of education
	Health	–	–	–
Department circular	Education	–	–	–
	Health	Joint Memorandum Circular No. 1, s. 2019 Measles Outbreak Response and Prevention of Transmission in Schools	2019/3/22	Department of health and department of education
		Department Circular No. 2019–0233 Adoption of the National Food and Waterborne Disease Prevention and Control Program Clinical Practice Guidelines on Acute Infections Disease Reference Manual	2019/6/14	Department of health
Department memorandum	Education	DepEd Memorandum No. 433, s. 2010 School Health Month Celebration	2010/10/7	Department of education
		DepEd Memorandum No. 101 Nutrition Month Celebration	2012/6/15	Department of education
		DepEd Memorandum No. 73, s. 2014 Nutrition Month Celebration	2014/6/26	Department of education
		DepEd Memorandum No. 82, s. 2015 Guidelines on the Implementation of School-Based Immunization Program	2015/7/31	Department of education
		DepEd Memorandum No. 100, s. 2016 Nutrition Month Celebration	2016/6/22	Department of education
		DepEd Memorandum No. 58, s. 2018 Nutrition Month Celebration	2018/4/23	Department of education
		DepEd Memorandum No. 148, s. 2018 Observance of the National Mental Health Week and the World Mental Health Day	2018/9/21	Department of education
		DepEd Memorandum No. 132, s. 2019 Observance of the National Mental Health Week and the World Mental Health Day	2019/10/7	Department of education
		DepEd Memorandum No. 194, s. 2016 Implementing Guidelines to DepEd Order No. 10, S. 2016 (Policy and Guidelines for Comprehensive Water, Sanitation and Hygiene in Schools Program)	2018/12/21	Department of education
		DepEd Memorandum No. 128, s. 2016 Implementation of School-Based Immunization Program	2016/7/16	Department of education
	Health	–	–	–
Analysis sheet 2 reviewed policy documents of the timeline of Early phase response (phase #2)				
Republic act	Education	Alternative Learning System Act (An Act Institutionalizing the Alternative Learning System in Basic Education for Out-Of-School Children in Special Cases and Adults and Appropriating Funds Therefor)	2020/12/23	18th Congress of the Philippines (senate and house of representatives)
	Health	Republic Act 11494 An Act Providing For Covid-19 Response and Recovery Interventions And Providing Mechanisms to Accelerate the Recovery and Bolster the Resiliency of the Philippine Economy, Providing Funds Therefor, and for Other Purposes	2020/07/27	18th Congress of the Philippines (senate and house of representatives)
Implementing rules and regulations	Education	–	–	–
	Health	2020 Revised Implementing Rules and Regulations of Republic Act No. 11332, or the Mandatory Reporting of Notifiable Diseases and Health Events of Public Health Concern Act	2020/8/27	
		Implementing Guidelines of Republic Act No. 11494, “An Act Providing For Covid-19 Response and Recovery Interventions And Providing Mechanisms to Accelerate the Recovery and Bolster the Resiliency of the Philippine Economy, Providing Funds Therefor, and for Other Purposes”	2020/11/3	(Not indicated)
Administrative order	Education	–	–	–
	Health	Administrative Order No. 2020–0015 Guidelines on the Risk-Based Public Health Standards for COVID-19 Mitigation	2020/4/27	Department of health
Proclamation	Education	–	–	–
	Health	Proclamation No.1253 Declaring November 29 to 01 December 2021 as Bayanihan, Bakunahan National COVID-19 Vaccination Days	2021/11/24	Office of the president
Department order	Education	DO 41, s. 2020 “Guidelines on the Implementation of the School Dental Health Care Program, Including Medical and Nursing Services for School Year 2020–2021”	2020/12/21	Department of education
		Department Order No. 031, s. 2021 Operational Guidelines on the Implementation of the School-Based Feeding Program	2021/8/9	Department of education
		DepEd Order No. 044, s. 2021 Policy Guidelines on the Provision of Educational Programs and Services for Learners with Disabilities in the K to 12 Basic Education Program	2021/11/2	Department of education
		Department Order No. 045, s. 2021 Policy Guidelines in the Selection of and Minimum Requirements for the Conversion of Certain Schools with Special Education (SPED) Centers into Prototype Inclusive Learning Resource Centers	2021/11/3	Department of education
	Health	–	–	–
Department circular	Education	–	–	–
	Health	Department Circular No. 2020- 0042 Interim Guidelines on 2019 Novel Coronavirus Acute Respiratory Disease (2019-nCoV ARD) Response in Schools & Higher Education Institutions	2020/2/5	Department of health
		Department Circular No. 0464, s. 2021 Interim Operational Guidelines on the COVID-19 Vaccination of the Pediatric Population Ages 12–17 Years Old with Comorbidities	2021/10/14	Department of health
Department memorandum	Education	Joint-Memo-003-s-2021 Comprehensive Sexuality Education—Adolescent Reproductive Health Convergence Pilot Implementation	2021/10/6	
		Memorandum OUCI-2020-307 Suggested Measures to Foster “Academic Ease” During the COVID-19 Pandemic	2020/10/30	Department of education
		DepEd Memorandum No. 058, s. 2020 Orientation for Regional and Schools Division Offices on Mental Health and Psychosocial Support Services in the Time of COVID-19 for Learners and DepEd Personnel	2020/7/1	Department of education
		DepEd Task Force COVID-19 Memorandum No. 82 Mental Health and Psychosocial Support Services for Learners, Parents and DepEd Personnel, and Printing of MHPSS Materials	2020/8/4	Department of education
		DepEd OUA Memorandum 1028-0198: Advisory on the Implementation of the Medical Nursing Services and ARH for SY 2020–2021	2020/10/19	Department of education
		DepEd Memorandum No. 023, s. 2020 Third Set of Policy Directives of DepEd Task Force COVID-19	2020/2/19	Department of education
		DepEd Memorandum No. 031, s. 2020 Fourth Set of Policy Directives of DepEd Task Force COVID-19	2020/3/5	Department of education
		DepEd Memorandum No. 034, s. 2020 Fifth Set of Policy Directives of DepEd Task Force COVID-19	2020/3/9	Department of education
		Bayanihan, Bakunahan National COVID-19 Vaccination Days	2021/11/25	Department of education
		Memorandum DM-OUCI-2021-055 Guidelines on the Counseling and Referral System of Learners for S.Y. 2020–2021	2021/03/03	Department of education
		DM-OUCI-2021-144—Implementation of Homeroom Guidance (HG) during Crisis Situation	2021/08/25	Department of education
		DepEd Memorandum No. 042, S. 2020 Guidelines for the Remainder of School Year 2019–2020 in Light of COVID-19 Measures	2020/3/15	Department of education
		DepEd Memorandum No. 060, s. 2021 2021 One Health Week Celebration as Part of Oplan Kalusugan sa DepEd	2021/9/7	Department of education
		DepEd Memorandum No. 071, s. 2021 Preparations for the Pilot Face-to-Face, Expansion and Transitioning to New Normal	2021/10/18	Department of education
		DepEd Memorandum No. 074, s. 2021 Inclusion and Promotion of Mental Health in all DepEd Events and Programs	2021/10/26	Department of education
		Department Memorandum No. 048, s. 2021 2021 Brigada Eskwela Implementing Guidelines	2021/8/2	Department of education
		DepEd DOH JMC No. 01, s. 2021 Operational Guidelines on the Implementation of Limited Face-to-Face Learning Modality	2021/9/27	Department of education, department of health
		CHED DOH JMC No. 2021-001 Guidelines on the Gradual Reopening of Campuses of Higher Education Institutions for Limited Face-to-face Classes during the COVID-19 Pandemic	2021/10/2	Department of health, commission on higher education
	Health	–	–	–
Other	Education	CHED Covid Advisory No. 5 Guidelines for the Prevention, Control, and Mitigation of the Spread of Coronavirus Disease 2019 (COVID-19) in Higher Education Institutions (HEIs)	2020/3/17	Commission on higher education
		Omnibus Guidelines on the Minimum Public Health Standards for the Safe Reopening of Institutions	2021/8/31	Department of health
	Health	Guidelines on the Pilot Implementation of Alert Levels System for COVID-19 Response in the National Capital Region	2021/9/13	Inter-agency task force
		Omnibus Guidelines on the Implementation of Community Quarantine in the Philippines	2020/5/15 2020/5/22 2020/6/3 2020/6/25 2020/7/2 2020/10/8 2020/10/15 2020/10/22 2020/11/19 2020/12/14 2021/1/14 2021/1/21 2021/2/11 2021/3/28 2021/4/3 2021/4/15 2021/5/6 2021/5/20 2021/8/6 2021/8/19	Inter-agency task force
		Memorandum From the Executive Secretary on the Implementation of Temporary Emergency Response And Recovery Measures Under Republic Act No. 11494, Otherwise Known As The Bayanihan To Recover As One Act	2020/8/23	Office of the president
		Memorandum from the Executive Secretary On Stringent Social Distancing Measures and Further Guidelines for the Management of the Coronavirus Disease 2019 (Covid-19) Situation	2020/3/13	Office of the president
		Memorandum from the Executive Secretary On Community Quarantine Over the Entire Luzon and Further Guidelines for the Management of the Coronavirus Disease 2019 (COVID-19) Situation	2020/3/16	Office of the president
Analysis sheet 3 reviewed policy documents of the timeline of chronic phase response/ recovery phase (phase #3)—Dec 15, 2021 onwards				
Executive order	Education	–	–	–
	Health	Executive Order No. 3, S. 2022 Allowing Voluntary Wearing Of Facemasks In Outdoor Settings And Reiterating The Continued Implementation Of Minimum Public Health Standards During The State Of Public Health Emergency Relative To The Covid-19 Pandemic		
		Executive Order No. 07 from the Office of the President entitled “Allowing Voluntary Wearing of Face Masks in Indoor and Outdoor Settings, Reiterating the Continued Implementation of Minimum Public Health Standards during the State of Public Health Emergency Relative to the COVID-19 Pandemic”	2022/11/03	Office of the president
Administrative order	Education	–	–	–
	Health	Joint Administrative Order No. 2022-0001 Guidelines on Healthy Settings Framework in Learning Institutions	2022/05/14	Department of health Department of social welfare and development Department of education Commission on higher education Legal education board Technical education and skills development authority Department of the interior and local government
Memorandum order	Education	CHED Memorandum Order No. 09, s. 2022 Updated Guidelines on the Implementation of Face to Face classes to Prevent and Mitigate COVID-19 Infections in Higher Education	2022/09/07	Commission on higher education
	Health	–	–	–
Memorandum circular	Education	Joint Memorandum Circular No. 001, s. 2022 Revised Operational Guidelines on the Progressive Expansion of Face to Face Learning Modality	2022/04/06	Department of education Department of health
Department order	Education	DO 010, s. 2022—Supplemental Guidelines on DepEd Order No. 031, s. 2021 (Operational Guidelines on the Implementation of the School-Based Feeding Program)	2022/03/11	Department of education
		DO No. 38, s. 2022—Supplemental Guidelines No. 2 to DepEd Order No. 031, s. 2021 (Operational Guidelines on the Implementation of the School-Based Feeding Program)	2022/09/09	Department of education
		DO No. 17, s. 2022—Guidelines on the Progressive Expansion of Face to Face Classes	2022/04/06	Department of education
		DO No. 23, s. 2022—Child Find Policy for Learners with Disabilities Towards Inclusive Education	2022/05/25	Department of education
		DO No. 12, s. 2022—Policy Guidelines for the Provision of Learning Resources and Needed Devices and Equipment and Funding Relevant Activities for the Implementation of Basic Education-Learning Continuity Plan		
		DO No. 1, s. 2022—Revised Policy Guidelines on Home Schooling Program		
	Health	–	–	–
Department circular	Education	–	–	–
	Health	Department Circular No. 2022–0610 Interim Operational Guidelines on the Implementation of Vaccination Activities during the Bakunahang Bayan II: Special COVID-19 Vaccination Days on December 5 to 7, 2022	2022/11/11	Department of health
		Department Circular No. 2022–0072 Interim Operational Guidelines on the Implementation of Vaccination Activities during the Bayanihan, Bakunahan National COVID-19 Vaccination Days, Part III on February 10–11, 2022	2022/2/7	Department of health
		Department Circular No. 2022–0131 Interim Operational Guidelines on the Implementation of Vaccination Activities during the Bayanihan, Bakunahan National COVID-19 Vaccination Days, Part IV (March to Vaccinate: Bringing COVID-19 Vaccines Closer to Homes, Communities, and Workplaces) on March 10–12, 2022	2022/3/4	Department of health
Department memorandum	Education	DM No. 26, s. 2022- Disability-Inclusive Teaching in Emergencies Online Course		Department of education
	Health	–	–	–

## Abbreviations

COVID-19: Coronavirus disease caused by the SARS-CoV-2 virus
AO: Administrative order
OK sa DepEd: Oplan Kalusugan sa Department of Education
SBFP: School-based feeding program
SDHP: School dental health care program
WASH: Water, sanitation, and hygiene
WinS: WASH in schools
FGD: Focus group discussions
KII: Key informant interview
UHC: Universal Health Care
SDO: Schools division office
